# Nutrition, functional status, polypharmacy, and digestive disturbances in institutionalized patients with dementia: insights from comprehensive screening at admission

**DOI:** 10.3389/fnut.2026.1779867

**Published:** 2026-07-02

**Authors:** Diogo Sousa-Catita, Paulo Mascarenhas, Miguel Grunho, Filipa Quaresma, Jorge Fonseca

**Affiliations:** 1 Egas Moniz Center for Interdisciplinary Research (CiiEM), Egas Moniz School of Health and Science, Almada, Portugal; 2 Residências Montepio, Serviços de Saúde S.A., Lisboa, Portugal; 3 GENE-Artificial Nutrition Team, Department of Gastroenterology, Hospital Garcia de Orta, Almada, Portugal; 4 Department of Neurology, Hospital Garcia de Orta, Almada, Portugal

**Keywords:** dementia, digestive disturbances, malnutrition, nutrition, polypharmacy

## Abstract

**Introduction:**

Malnutrition, loss of muscle mass, functional decline, and polypharmacy are highly prevalent in people with dementia, contributing to accelerated frailty, dependency, and institutionalization. Early multidimensional assessment at the time of admission to long-term care units is essential for identifying nutritional and functional vulnerabilities and guiding timely, individualized interventions. This study aimed to characterize the nutritional, anthropometric, and functional status of patients with dementia and to examine the association between digestive disturbances and polypharmacy.

**Methods:**

A multicenter cross-sectional study was conducted, including adults diagnosed with dementia. Nutritional status was assessed using the Mini Nutritional Assessment (MNA^®^) and the Global Leadership Initiative on Malnutrition (GLIM) criteria. Anthropometric evaluation included body mass index (BMI), mid-upper arm circumference (MUAC), triceps skinfold thickness (TSF), mid-arm muscle area (MAMA), and calf circumference (CC). Functional status was evaluated using handgrip strength and the SARC-F questionnaire for sarcopenia risk. Cognitive status, medication profiles, and digestive symptoms were systematically recorded. Multivariate regression models were applied to examine factors associated with anthropometric indicators of muscle mass and digestive symptoms.

**Results and conclusion:**

Malnutrition was highly prevalent, with nearly all 112 participants classified as malnourished or at nutritional risk according to MNA^®^, and 79.5% fulfilling GLIM criteria. Severe loss of muscle mass and marked functional impairment were observed, and more than 85% of participants were identified as being at risk of sarcopenia according to SARC-F. Polypharmacy affected over 90% of patients, and each additional prescribed medication was associated with approximately 23% higher odds of digestive disturbances. Anthropometric muscle mass indicators were independently associated with sex and BMI, whereas functional measures reflected physical performance rather than muscle mass. These findings highlight the need for systematic, multidomain assessment integrating nutritional screening, anthropometric and functional evaluation, gastrointestinal symptom assessment, and medication review to support early, targeted, and interdisciplinary interventions for people with dementia entering long-term care units.

## 1 Introduction

Dementia is a heterogeneous neurodegenerative syndrome characterized by progressive cognitive decline, behavioral disturbances, and impaired capacity for activities of daily living. It represents a major public health challenge in aging populations, affecting an estimated 55 million people worldwide, a figure projected to triple by 2050 due to demographic aging and increased life expectancy (1). Alzheimer’s disease (AD) is the most prevalent subtype, a tau pathology driven primarily by amyloid-β accumulation and synaptic dysfunction (2). Vascular dementia (VaD) arises from cerebrovascular lesions and impaired cerebral perfusion. In contrast, dementia with Lewy bodies (DLB) is associated with α-synuclein aggregation, and frontotemporal dementia (FTD) involves selective degeneration of frontal and temporal lobes (3). Each of these most frequent subtypes exerts distinct effects on cognition, behavior and, most notably, on nutritional status, due to differences in appetite regulation, motor function, and behavioral symptomatology (4).

Nutritional status is increasingly recognized as both a determinant of dementia progression and an independent risk factor for adverse outcomes. Malnutrition, sarcopenia, and frailty are highly prevalent in this population, contributing to accelerated functional decline, increased susceptibility to infections, and higher morbidity and mortality (5). Cognitive impairment can compromise dietary intake through forgetfulness, altered taste perception, swallowing difficulties, and behavioral disturbances such as agitation or food refusal (6). Conversely, inadequate nutrition may exacerbate neurodegenerative processes through mechanisms including chronic inflammation, oxidative stress, and micronutrient deficiencies, suggesting a bidirectional relationship between cognitive decline and malnutrition (7). Nutritional impairment increases dementia progression and may not be reversible. In advanced dementia, enteral feeding is generally not recommended according to current ESPEN guidance, although individualized decisions may be considered in highly selected clinical situations (8, 9).

The assessment of nutritional risk in older adults with dementia requires a multidimensional approach. There are concerns that traditional anthropometric measures, such as body mass index (BMI) and mid-upper arm circumference (MUAC), may underestimate malnutrition due to age-related changes in body composition and fluid distribution (10). Therefore, validated tools such as Nutritional Risk Screening 2002 (NRS-2002), Mini Nutritional Assessment (MNA^®^), Sarcopenia Screening Tool (SARC-F), and Global Leadership Initiative on Malnutrition (GLIM) criteria are critical for early detection of nutritional decline and functional impairment (11–13). Integrating these instruments at admission to health institutions allows clinicians to identify at-risk individuals and implement personalized interventions, potentially mitigating further cognitive and physical impairment.

Polypharmacy is another critical factor affecting nutritional status in older adults with dementia. The use of multiple medications, including psychotropics, anticholinergics, and cardiovascular drugs, can contribute to anorexia, dysgeusia, digestive disturbances, and micronutrient deficiencies (14). Polypharmacy may also increase the risk of drug–nutrient interactions, further compromising dietary intake and absorption, and thereby amplifying vulnerability to malnutrition and sarcopenia (15). Identifying the impact of polypharmacy is essential, as it may modulate the effects of both cognitive decline and comorbidities on dietary intake.

Despite increasing recognition of the interplay among dementia, polypharmacy, and nutrition, there remains a scarcity of comprehensive studies that combine demographic, anthropometric, and functional assessments with the simultaneous application of multiple validated screening tools in institutionalized populations. Understanding the relationships among dementia, nutritional risk, polypharmacy, and sarcopenia is essential for delivering person-centered care, optimizing resource allocation, and informing the development of evidence-based clinical guidelines.

The present study aims to provide a detailed characterization of patients with dementia upon admission to long-term care units, integrating demographic and anthropometric data with comprehensive assessments of nutritional status, sarcopenia, and functional capacity. Specifically, this investigation seeks to:

(1) Assess the prevalence of malnutrition and sarcopenia in this population;

(2) Evaluate anthropometric and functional parameters (body mass index (BMI), mid-upper arm circumference (MUAC), triceps skinfold thickness (TSF), mid-arm muscle area (MAMA), calf circumference (CC), and handgrip strength (HGS)] as feasible screening markers of nutritional and functional vulnerability at admission;

(3) Examine the associations between nutritional status, anthropometric and functional measures, and relevant clinical features, including polypharmacy and digestive disturbances;

(4) Explore factors associated with anthropometric indicators of muscle mass and with the presence of digestive symptoms in patients with dementia at institutional admission.

By adopting a multidimensional approach, this study aims to contribute to a deeper understanding of the complex interactions between dementia, polypharmacy, nutritional status, and functional decline, ultimately supporting the design of targeted interventions to improve clinical outcomes and quality of life in this vulnerable population.

## 2 Materials and methods

### 2.1 Study design and setting

This observational, cross-sectional study was conducted in four Portuguese long-term Care Units (LTCU) of the metropolitan area of Lisbon. The study complied with the principles of the Declaration of Helsinki and was approved by the Institutional Ethics Committee of Egas Moniz School of Health and Science (approval number 1124). Written informed consent was obtained from the legal representatives of all participants prior to enrolment.

### 2.2 Participants

A convenience sample was obtained, encompassing all consecutive admissions to the LTCU between 20 December 2023 and 30 May 2025. Inclusion criteria were: (i) a confirmed diagnosis of dementia documented in medical records; (ii) age ≥ 18 years; and (iii) availability of complete demographic, anthropometric, and clinical data at admission. Patients were excluded if they presented with acute medical instability or were in a terminal stage of illness. Informed consent was obtained from both patients and their legal representatives, and enrollment was contingent upon the agreement of all parts.

A total of 124 patients were initially screened. Twelve were excluded due to incomplete data (*n* = 9) or acute clinical instability (*n* = 3), resulting in a final sample of 112 participants included in the analysis.

### 2.3 Demographic and clinical data

Sociodemographic variables collected included age, sex, education level (years of formal schooling), and ethnicity. Clinical information comprised dementia subtype (Alzheimer’s disease, vascular dementia, dementia with Lewy bodies, frontotemporal dementia, mixed dementia, or unspecified dementia).

The diagnosis of dementia was established clinically by a neurologist or psychiatrist prior to study enrollment, in accordance with the most appropriate and well-established diagnostic criteria [e.g., National Association Workgroup Institute on Aging–Alzheimer’s (NIA-AA) (16), International Statistical Classification of Diseases and Related Health Problems, Tenth Revision (ICD-10) (17), or Diagnosis and Statistical Manual of Mental Disorders, Fifth Edition (DSM-5) (18)]. Cognitive status was assessed using the Mini-Mental State Examination (MMSE) (19) and staged according to the Global Deterioration Scale (GDS) (20). Notably, since MMSE is highly sensitive to memory loss, but far less sensitive to other cognitive impairments (such as executive function, planning, and processing speed), a normal MMSE score in specific participants did not preclude a clinical diagnosis of dementia, particularly in early-stage disease or subtypes with preserved memory but significant functional or behavioral impairment across other neurocognitive domains (e.g., vascular dementia).

Additionally, the medication profile and the presence of gastrointestinal symptoms were recorded, as these digestive disturbances frequently interfere with the patient’s feeding.

### 2.4 Anthropometric data

Body weight was measured to the nearest 0.1 kg using a calibrated digital scale, and height to the nearest 0.1 cm using a stadiometer. In cases where direct measurement was not feasible due to mobility constraints, weight was estimated using validated predictive equations proposed by Rabito et al. (21). BMI was calculated as weight (kg) divided by height^2^ (m^2^). If patients were bedridden and unable to stand for weight and height measurements, BMI was estimated using MUAC and regression equations described by Powell-Tuck and Hennessy (22). This method has been used previously and has been shown to provide reliable BMI estimates (23). MUAC, calf circumference (CC), and triceps skinfold thickness (TSF) were measured according to ISAK standards (24), using a non-elastic tape for circumferences and a Harpenden skinfold caliper for TSF.

MAMA was calculated from MUAC and TSF using the following formula (25):


$$\mathrm{MAMA}\left({\mathrm{cm}}^{2}\right)=\frac{{\left[\mathrm{MUAC}\,\left(\mathrm{cm}\right)-\left(\pi \,x\,\mathrm{TSF}\,\left(\mathrm{cm}\right)\right)\right]}^{2}}{4\,\pi })$$

MAMA provides an estimate of skeletal muscle reserve. Values were interpreted according to Frisancho’s reference standards:

<5th percentile → Severe muscle depletion.

5th–15th percentile → Moderate muscle depletion.

≥15th percentile → Normal muscle status.

Low muscle mass was initially defined as CC below 31 cm (26). MAMA and TSF were subsequently assessed to provide an additional characterization of body composition. While MUAC reflects the combined contributions of muscle, subcutaneous fat, and bone tissue, MAMA, derived from MUAC and TSF, offers a more specific estimate of peripheral lean tissue reserves. It is therefore a more appropriate anthropometric proxy for skeletal muscle mass (25).

Mid-upper arm circumference cut-offs were applied according to ESPEN recommendations, with values < 22 cm in women and <23 cm in men indicating an increased risk of undernutrition (27). TSF was interpreted using Frisancho reference data, with values below the 5th percentile for age and sex considered indicative of subcutaneous fat depletion (25). This combined approach allowed discrimination among global arm size, adipose tissue reserves, and muscle mass depletion, improving the accuracy of nutritional and sarcopenia-related assessments.

To account for age-related changes in body composition, BMI classification followed Lipschitz criteria (28), which apply age-specific cut-off points for undernutrition in older adults (Table 1).

**TABLE 1:** Body mass index (BMI) classification according to age.

Age group	Low	Normal	High
<65 years	<18.5 kg/m^2^	≥18.5 and <25 kg/m^2^	≥25 kg/m^2^
≥65 years	<22 kg/m^2^	≥22 and <27 kg/m^2^	≥27 kg/m^2^

All assessments were conducted at admission by trained healthcare professionals (dietitians) as part of routine clinical evaluation in the participating long-term care units, following standardized procedures across centers. Data was subsequently extracted for research purposes.

### 2.5 Muscle strength

Handgrip strength was assessed using a calibrated dynamometer (Jamar^®^). Measurements were performed by trained healthcare professionals (nutritionists or nurses) following a standardized protocol, with patients seated (or in the most appropriate position when bedridden), the elbow flexed at 90°, and standardized verbal encouragement provided. When feasible, two attempts were performed on the dominant hand. However, only the first valid attempt was systematically recorded across all participating units and was therefore used for analysis.

Although EWGSOP2 recommends using the highest value from repeated measurements, this was not possible in the present study because repeated measurements were not consistently available for most participants. The use of the first valid attempt was therefore a pragmatic methodological decision in the context of advanced dementia, reduced cooperation, fatigue, and difficulty following repeated instructions.

This approach was adopted because the study population included individuals with moderate-to-severe cognitive impairment (mean MMSE = 11). In this context, repeated measurements may be influenced by reduced attention span, fatigue, apathy, and difficulty in consistently following instructions, potentially leading to inconsistent or non-reproducible performance across attempts rather than true maximal effort. Therefore, the first measurement was considered the most reliable and representative estimate of voluntary effort under standardized conditions in this population, while acknowledging that it was the possible adaptation of the maximal grip strength as defined by EWGSOP2 recommendations (29). Low HGS was defined according to EWGSOP2 thresholds (<27 kg for men and <16 kg for women) (29).

### 2.6 Nutritional assessment

Nutritional status was evaluated at admission using the MNA^®^ (11), a tool primarily validated for individuals aged 65 years and older. In this study, the MNA^®^ was also applied to patients with dementia younger than 65 years to ensure methodological consistency and comparability across the entire sample. This approach aligns with our previous research, where the application of the MNA^®^ in similar age ranges did not compromise the validity or reliability of findings (30). The MNA^®^ was used as the initial nutritional risk screening tool prior to the application of the GLIM diagnostic criteria.

Diagnostic confirmation of malnutrition followed the GLIM framework, which integrates phenotypic indicators (weight loss, low BMI, and reduced muscle mass) with etiologic components (reduced intake and disease burden/inflammation) (13). For a GLIM-based diagnosis, the presence of at least one phenotypic criterion and one etiologic criterion was required.

Phenotypic criteria were operationalized as unintentional weight loss, low BMI according to age-specific cut-offs, or reduced muscle mass (assessed by CC < 31 cm and/or MAMA below the 15th percentile). Regarding etiologic criteria, chronic dementia was operationalized as fulfilling the GLIM disease-burden criterion in the primary analysis, given its established association with functional dependency, reduced intake, inflammatory vulnerability, and nutritional decline. However, because all participants had dementia, this operational decision meant that GLIM classification was primarily driven by the presence of phenotypic criteria.

Reduced food intake was clinically assessed when reliable information was available from medical records or caregiver reports; however, this data were not systematically complete for all participants. Therefore, a sensitivity analysis using reduced food intake as the sole etiologic criterion was not feasible, and this methodological dependency was explicitly considered when interpreting GLIM-based malnutrition prevalence.

### 2.7 Sarcopenia risk

Risk of sarcopenia was screened using the SARC-F questionnaire, with scores ≥ 4 indicating probable sarcopenia (31).

### 2.8 Polypharmacy assessment

Medication data were collected from formalized prescribing and administration records available in the medical charts at the time of admission and complemented, when necessary, by review of clinical documentation. These data reflect the prescribed and administered medication profile recorded at institutional admission. However, independent verification of actual medication intake before admission was not available, and recent medication changes or non-adherence prior to institutionalization could not be fully excluded.

Polypharmacy was defined as the concurrent use of five or more prescribed medications, including psychotropics, cardiovascular agents, and other drugs for chronic conditions (32). Medications were categorized into therapeutic groups based on their primary clinical indication. Specifically, the “Incontinence” group included antimuscarinic and beta-3 agonist agents, while the “Genitourinary dysfunction” group primarily comprised treatments for prostatic hypertrophy (e.g., alpha-blockers). The “Anti-anemic” group consisted exclusively of oral iron and vitamin replacements (e.g., B12, folic acid).

A formal Anticholinergic Cognitive Burden (ACB) score could not be validly computed because the extracted dataset contained medication counts and broad therapeutic classes, but not consistently coded individual active substances for all participants. Since ACB scoring requires drug-level classification, assigning ACB values at the class level would introduce substantial exposure misclassification. Therefore, the analysis was restricted to total medication count and therapeutic classes, and the absence of a validated anticholinergic burden measure was considered an important limitation.

### 2.9 Digestive symptoms

Digestive symptoms were systematically assessed due to their recognized influence on dietary intake and nutritional status in patients with dementia. Constipation, diarrhea, dysphagia, nausea, vomiting, bloating, abdominal discomfort, and loss of appetite were recorded through medical records and clinical observation.

Digestive symptoms were coded as a binary variable (present/absent) for statistical analysis.

### 2.10 Statistical analysis

Given the observational and exploratory nature of this study, a formal a priori sample size calculation was not performed. A consecutive sampling approach was adopted, including all eligible patients assessed during the recruitment period. The final sample size therefore reflects the available real-world population admitted to the participating long-term care units and should be considered when interpreting smaller effect sizes and exploratory multivariable analyses.

All statistical analyses were conducted using Python (version 3.12; pandas 2.2, numpy 1.26, scipy 1.12, statsmodels 0.14). Continuous variables were summarized as mean ± standard deviation (SD). Categorical variables were reported as frequencies (%) with 95% confidence intervals when relevant.

Pairwise associations between SARC-F, HGS, and muscle mass indicators (MAMA and CC) were explored using both Pearson’s (r) and Spearman’s (ρ) correlation coefficients. Correlation strength was interpreted as weak (|r| < 0.30), moderate (0.30–0.49), and strong (≥0.50). Multivariable linear regression models with HC3-robust standard errors were fitted, with MAMA and CC as the dependent variables and SARC-F and HGS as the main independent variables of interest. Covariates included in multivariable models (age, sex, BMI, GLIM classification, and GDS stage) were selected a priori based on clinical relevance and existing literature on nutritional status and sarcopenia in dementia, rather than solely on statistical criteria. Model assumptions (linearity, normality, and homoscedasticity) were verified graphically; inspection of residual-versus-fitted plots and Q–Q plots confirmed approximate normality and homoscedasticity. Variance inflation factors (VIFs) were inspected to exclude multicollinearity.

To compare nutritional and anthropometric measures across medication groups (≤4, 5–10, >10 medications), Kruskal–Wallis tests were applied, followed by pairwise Mann–Whitney U tests with Holm–Bonferroni correction for multiple comparisons when applicable.

For the categorical GLIM variable, associations with medication group were examined using Chi-square tests of independence, with Cramér’s V as the effect size. As a complementary analysis, Spearman rank correlations were computed between the raw number of medications and continuous nutritional parameters (BMI, MUAC, MAMA, CC, TSF, HGS, and MNA^®^). All tests were two-tailed, and *p*-values < 0.05 were considered statistically significant.

To explore the relationship between polypharmacy and gastrointestinal (GI) disturbances, a binary logistic regression model was developed. The dependent variable indicated the presence or absence of digestive symptoms (coded 1 = present; 0 = absent), including constipation, diarrhea, dysphagia, nausea, vomiting, bloating, abdominal discomfort, and loss of appetite. These symptoms were identified through clinical records, caregiver reports, and direct assessment at admission.

The primary independent variable was the total number of prescribed medications, reflecting polypharmacy burden. Additional covariates included the GLIM nutritional classification (no, moderate, or severe malnutrition) and dementia severity, as assessed by the Global Deterioration Scale (GDS). All categorical predictors were dummy-coded. Multicollinearity was evaluated using the variance inflation factor (VIF); values < 3.0 were considered acceptable.

Pre-specified full models were initially fitted including clinically relevant covariates selected a priori based on biological plausibility and previous literature. Reduced models were subsequently presented only when removal of covariates did not materially change the main association, coefficient stability, or clinical interpretation. Model diagnostics included inspection of residual plots, Q–Q plots, variance inflation factors, adjusted R^2^, RMSE, and information criteria where applicable. Full adjusted outputs are provided in the Supplementary material to ensure transparency.

The primary analytical focus of the study was the characterization of nutritional status at admission and the association between polypharmacy burden and digestive symptoms. Analyses involving MAMA, CC, HGS, and SARC-F were conducted as complementary exploratory analyses to better describe structural and functional vulnerability in this population.

In the multivariable linear regression models, age, sex, and BMI were included as primary covariates to adjust for fundamental biological determinants of muscle mass and strength. Although cognitive impairment (MMSE) and nutritional status (MNA^®^) are clinically relevant, they were not included simultaneously in the final models to avoid potential multicollinearity and model overfitting. Specifically, the MNA^®^ incorporates BMI and functional components, while MMSE scores in this population are strongly associated with functional dependence (SARC-F). Including these overlapping variables could destabilize the models and inflate standard errors, thereby complicating the interpretation of independent associations.

To ensure full transparency and reproducibility, complete outputs for all multivariable models, including unstandardized and standardized coefficients, 95% confidence intervals, VIF values, and model fit indices, as well as comprehensive Spearman correlation matrices, are provided as Supplementary Tables 1–3.

### 2.11 Data availability

The datasets generated and analyzed during the current study are not publicly available due to clinical data from the enrolled units requiring authorization for consultation. Still, they are available from the corresponding author on reasonable request.

## 3 Results

### 3.1 Demographic and clinical characteristics

A total of 112 patients were included, with a predominance of females (58.9%). The cohort was characterized by advanced age (mean 82.6 ± 9.3 years), with most participants being Caucasian (99.1%).

The mean time since dementia diagnosis was 3.94 ± 2.71 years, with nearly half of the patients presenting a disease duration longer than 3 years. Unspecified dementia was the most frequent subtype (59.8%), followed by Alzheimer’s disease (24.1%) and vascular dementia (13.4%) (Table 2).

**TABLE 2:** Demographic and clinical characteristics of the study population.

Variable	*n* (%) or mean ± SD
Female	66 (58.9)
Age (years)	82.6 ± 9.3
Caucasian ethnicity	111 (99.1)
Time since diagnosis	3.9 (2.71)
Dementia type	
Alzheimer’s disease	27 (24.1)
Vascular dementia	15 (13.4)
Mixed dementia	2 (1.8)
Dementia with Lewy bodies	1 (0.9)
Unspecified dementia	67 (59.8)

### 3.2 Anthropometric profile

The anthropometric characteristics of the cohort are summarized in Table 3. Overall, a high prevalence of undernutrition and marked alterations in body composition were observed, with 45.5% of participants classified as underweight according to BMI.

**TABLE 3:** Anthropometric characteristics.

Parameter	Mean ± SD	Total low score (%)
Weight (kg)	60.2 ± 13.9	–
Height (m)	1.60 ± 0.09	–
BMI (kg/m^2^)	23.5 ± 4.6	45.5
MUAC (cm)	24.6 ± 3.6	22.3
CC (cm)	29.6 ± 4.2	34.8
TSF (mm)	13.4 ± 6.0	21.4
MAMA (cm^2^)	33.7	75

Body composition analysis revealed a predominance of muscle depletion, with 75% of patients presenting moderate-to-severe reduction in MAMA. CC further supported this finding, with over one-third of participants exhibiting values indicative of low muscle mass.

Adipose tissue depletion was also frequent, as reflected by TSF measurements, although less pronounced than muscle loss. Sex-related differences were evident, with men showing a higher prevalence of severe muscle depletion, while women more frequently exhibited fat mass reduction. Detailed values are presented in Table 3.

### 3.3 Muscle strength and sarcopenia risk

Functional impairment was highly prevalent in this cohort. HGS values were markedly reduced, with most participants classified below EWGSOP2 thresholds, particularly among males. The prevalence of low HGS was high (88.4%); however, this estimate should be interpreted as exploratory. Because only the first valid attempt was systematically available, and because advanced cognitive impairment may affect comprehension, cooperation, and reproducibility of effort, this value may overestimate the prevalence of low muscle strength relative to the standard EWGSOP2 protocol.

Consistently, 86.6% of patients were identified as being at risk of sarcopenia according to SARC-F (Table 4).

**TABLE 4:** Functional and sarcopenia-related parameters.

Parameter	*n* (%) or mean ± SD
HGS (kg)	11.3 ± 6.5
Low handgrip (EWGSOP2)	99 (88.4)
SARC-F ≥ 4 (risk)	97 (86.6)

Correlational analyses showed weak associations between SARC-F and CC (*r* = −0.24; *p* < 0.05) and between HGS and CC (*r* = 0.24; *p* < 0.05). In the initial multivariable models, age, sex, BMI, HGS, and SARC-F were considered as candidate predictors. In the final adjusted models, neither SARC-F nor HGS emerged as independent predictors of MAMA or CC. Instead, male sex and higher BMI were independently associated with muscle mass, explaining approximately 44% of the variance in MAMA. Detailed outputs for the linear regression models are provided in Supplementary Table 1.

### 3.4 Nutritional status

Nutritional assessment revealed a very high burden of malnutrition in this population (Table 5). The median MNA^®^ score was 17, with almost all participants (99.1%) classified as malnourished or at risk.

**TABLE 5:** Nutritional assessment.

Tool	Category	*n* (%)
MNA^®^	Malnourished	53 (47.3)
	At risk of malnutrition	58 (51.8)
	Normal	1 (0.9)
GLIM	No malnutrition	23 (20.5)
	Moderate malnutrition	52 (46.4)
	Severe malnutrition	37 (33.0)

According to GLIM criteria, 79.5% of patients were diagnosed with malnutrition, including 46.4% with moderate and 33.0% with severe malnutrition, confirming the severity of nutritional impairment in this cohort.

### 3.5 Cognitive status

The cohort exhibited marked cognitive impairment, with a mean MMSE score of 11 points. According to GDS classification, most patients were in moderate to moderately severe stages of dementia, with no participants in the absence of cognitive decline (stage 1), reflecting the advanced clinical profile of this population.

### 3.6 Polypharmacy

**FIGURE 1:**
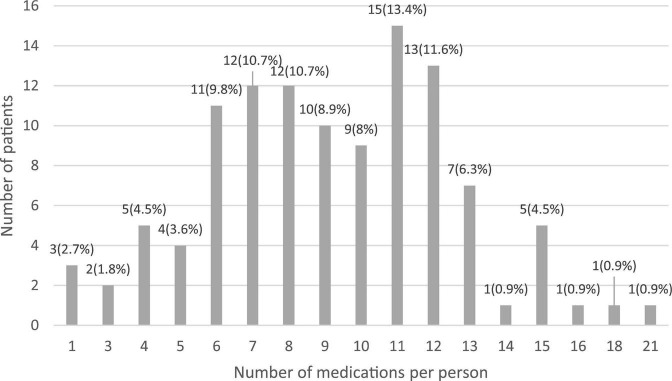
Number of medications per patient.

Polypharmacy was highly prevalent, affecting 91% of participants (Figure 1). The distribution of medications by pharmacological class is detailed in Table 6, with particularly high use of antidepressants, antihypertensives, antipsychotics, and lipid-lowering agents.

**TABLE 6:** Frequency and percentage of patients by drug class.

Drug class	*n*	%
Antidepressant	75	67
Antihypertensive	63	56.3
Antipsychotic	59	52.7
Antidyslipidemic	55	49.1
Antidementia	54	48.2
Antacid/antiulcer	51	45.5
Analgesic/antipyretic	38	33.9
Laxative	37	33
Anticoagulant	32	28.6
Anxiolytic, sedative and hypnotic	31	27.7
Antiplatelet	29	25.9
Diuretic	28	25
Antidiabetic	28	25
Genitourinary dysfunction	25	22.3
Vitamin	25	22.3
Antianemic	24	21.4
Antiparkinsonian	16	14.3
Anticonvulsant	15	13.4
Thyroid hormones	14	12.5
Narcotic analgesic	13	11.6
Antiepileptic	12	10.7
Bronchodilator	10	8.9
Antigout	9	8
Vasodilator	7	6.3
Corticosteroids	6	5.4
Antihistamine	5	4.5
Antibiotic	5	4.5
Antiarrhythmic	4	3.6
Incontinence	2	1.8
Antiglaucoma	2	1.8
Antifungal	2	1.8
Antineoplastic	2	1.8
Mucolytic	1	0.9
Muscle relaxant	1	0.9

No statistically significant differences were observed across medication groups for anthropometric or nutritional parameters, including GLIM classification (all *p* > 0.05). Detailed statistical associations are presented in Table 7.

**TABLE 7:** Associations between polypharmacy and nutritional/functional parameters.

Parameter	Correlation with number of drugs (ρ)	*P*-value	Comparison: <5 vs. >10 drugs (mean ± SD)	*P*-value*
Nutritional status				
MNA^®^ score	−0.084	0.381	10.2 ± 3.1 vs. 9.8 ± 2.8	0.612
BMI (kg/m^2^)	0.012	0.901	23.1 ± 4.2 vs. 23.8 ± 4.5	0.784
Muscle mass (indirect)				
CC (cm)	0.045	0.642	30.2 ± 3.5 vs. 31.1 ± 3.8	0.521
MAMA (cm^2^)	0.092	0.335	32.4 ± 8.1 vs. 34.2 ± 9.2	0.415
Muscle strength				
HGS (kg)	**0.225**	**0.017**	**6.6 ± 4.2 vs. 12.7 ± 5.8**	**0.045**

*ρ, Spearman’s rank correlation coefficient. *P*-value for between-group comparison (Mann-Whitney U test). Significant values (*p* < 0.05) are highlighted in bold.

Handgrip strength differed across medication groups (*p* = 0.045) and showed a weak positive correlation with the number of medications (ρ = 0.225; *p* = 0.017). Participants taking more than 10 medications had higher HGS than those taking fewer than 5 medications. The complete correlation matrix is presented in Supplementary Table 2.

### 3.7 Digestive symptoms

Digestive disturbances were present in 50.9% of patients, underscoring their high prevalence in this institutionalized dementia population.

In the multivariable logistic regression model, the number of medications was independently associated with the presence of digestive disturbances. The final model was obtained after stepwise exclusion of non-significant variables, namely nutritional status (GLIM criteria) and dementia severity (GDS stage). Each additional prescribed medication was associated with a 23% higher odds of presenting digestive symptoms (OR = 1.23; 95% CI: 1.09–1.39; *p* = 0.001).

No evidence of multicollinearity was observed among the variables included in the analysis. Detailed logistic regression analysis results are presented in Supplementary Table 3.

## 4 Discussion

The long-term care units (LTCUs) included in this multicenter study primarily admit older adults characterized by advanced dementia, profound functional dependency, and a high burden of comorbidities, frequently following acute hospitalizations. These patients typically require assistance with activities of daily living and present complex, interrelated clinical and nutritional needs that demand specialized care.

This study provides a comprehensive evaluation of the nutritional and functional landscape of patients with dementia upon institutional admission, integrating detailed anthropometric profiling, muscle strength assessment, and the simultaneous application of validated screening and diagnostic tools (MNA^®^, GLIM, and SARC-F). Our findings reveal an alarmingly high prevalence of malnutrition and functional impairment, with polypharmacy being nearly universal, and digestive disturbances affecting most patients. These results underscore the multidimensional vulnerability of this population and reinforce the critical need for systematic, multidomain assessments at the point of institutional admission to guide early, targeted, and interdisciplinary interventions.

### 4.1 High burden of malnutrition

In the present study, all but one patient were malnourished or at risk according to MNA^®^, and 79.5% fulfilled GLIM criteria for malnutrition, with one-third classified as severely malnourished. These proportions exceed those generally reported in institutionalized older populations (30%–60%) (33, 34).

This high prevalence should be interpreted with caution, as it is partly influenced by the operationalization of the GLIM criteria in this cohort. In accordance with the GLIM framework, dementia was considered to fulfill the etiologic criterion of disease burden/inflammation for all participants (13). Consequently, the diagnosis of malnutrition relied primarily on phenotypic criteria, which may have increased sensitivity at the expense of specificity compared with approaches requiring more restrictive etiologic confirmation, such as documented reduced food intake or acute inflammatory conditions. Therefore, the prevalence observed with GLIM may, in part, reflect this methodological dependency. However, the results obtained with the MNA^®^, indicating an even higher prevalence of malnutrition/risk of malnutrition than the prevalence found with the GLIM, confirm the impairment of nutritional status.

Beyond methodological considerations, several clinical factors likely contributed to the high burden of malnutrition. Advanced age (mean 82.6 years), severe cognitive impairment (mean MMSE of 11 points), and marked functional dependence are well-established determinants of poor nutritional status. Dementia-related impairments interfere with appetite regulation, feeding autonomy, and dietary quality, often leading to inadequate intake and a preference for energy-dense, nutrient-poor foods (35, 36). In addition, the timing of assessment at admission to long-term care may capture patients at a stage when nutritional decline is most pronounced. Nevertheless, previous studies suggest that nutritional status may partially improve following institutionalization, as structured feeding support and dietary supervision can help stabilize weight and attenuate further muscle loss in people with dementia (8).

Anthropometric indicators provided valuable complementary information, although they demonstrated lower sensitivity for detecting malnutrition compared with MNA^®^ or GLIM. MUAC identified only 22.3% of participants as undernourished, reflecting its limited discriminatory capacity in this context (11). This with the composite nature of MUAC, which reflects both lean and adipose tissue and finding is consistent may therefore mask selective muscle depletion in advanced dementia. Despite this limitation, may MUAC remain clinically useful due to its simplicity, reproducibility, and prognostic value in frail older adults (37), particularly for longitudinal monitoring.

Distinct depletion patterns were observed across tissue compartments. TSF indicated reduced adipose reserves (≤15th percentile) in most participants, while MAMA providing a more specific estimate of peripheral lean tissue, confirmed that muscle mass loss predominated over fat depletion, with 75% of participants showing moderate-to-severe depletion, particularly among men (38). These findings suggest that loss of lean mass, rather than generalized body size reduction, is the principal driver of malnutrition in this population. CC supported this interpretation, with 34.8% of participants presenting values < 31 cm, consistent with reduced muscle mass and functional impairment (23). Taken together, MAMA and CC emerged as more sensitive indicators of sarcopenic malnutrition, showing closer alignment with functional measures (HGS and SARC-F) than MUAC or TSF alone, which primarily reflect mixed or adipose compartments (39).

Interestingly, these findings contrast with previous observations from our group in dysphagic patients, in whom fat depletion (TSF) was more pronounced, suggesting a relative preservation of lean tissue as a potential adaptive response (40). In contrast, the present cohort demonstrates a predominance of muscle wasting, consistent with the catabolic and inactivity-related mechanisms underlying advanced dementia.

In long-term care settings, patients are frequently admitted at advanced stages of dementia, often without etiological specification due to diagnostic uncertainty (41). Although dementia subtype may influence nutritional patterns (e.g., anorexia and apathy in Alzheimer’s disease versus dysphagia and rigidity in Parkinsonian syndromes) (35, 36), the predominance of unspecified dementia (60%) in this sample limits subtype-specific analyses. Therefore, shared nutritional vulnerabilities should be prioritized in clinical practice, regardless of the underlying etiology.

### 4.2 Sarcopenia and functional decline

Recent evidence supports a strong bidirectional relationship between sarcopenia and cognitive decline, both contributing to increased frailty, functional dependency, and mortality (42). In the present cohort, functional impairment was particularly pronounced, with 86.6% of participants classified as at risk of sarcopenia according to SARC-F and 88% exhibiting low HGS, with mean values markedly below EWGSOP2 thresholds (43–46).

Notably, HGS was positively associated with CC but not with MAMA, suggesting that peripheral muscle performance may decline independently of total muscle area. Similarly, SARC-F scores were inversely associated with CC but not with MAMA, and neither measure independently predicted muscle mass in adjusted models. These findings reinforce the concept that functional measures and anthropometric/morphologic indicators capture distinct dimensions of sarcopenia. This interpretation is consistent with previous evidence indicating that, in advanced dementia, impairments in muscle quality and neuromuscular coordination may precede measurable reductions in muscle mass (47).

The coexistence of marked functional impairment and muscle depletion highlights the importance of integrating both structural (MAMA and CC) and performance-based (HGS and SARC-F) assessments in this population. Cognitive decline, immobility, and sarcopenia are likely to interact synergistically, accelerating the trajectory toward frailty, dependency, and adverse clinical outcomes.

These findings suggest that functional measures such as SARC-F and HGS primarily reflect physical performance rather than muscle quantity in patients with advanced dementia.

### 4.3 Polypharmacy and digestive disturbances as nutritional risk factors

In the present study, polypharmacy was nearly universal (91.1%) and represents a critical, yet potentially modifiable, factor associated with nutritional status (48). Psychotropic, anticholinergic, and cardiovascular agents may reduce appetite, impair swallowing, alter gastrointestinal motility, or cause xerostomia, thereby contributing to the exacerbation of malnutrition and sarcopenia (49, 50). In particular, medications with anticholinergic properties are known to impair gastrointestinal motility and secretion, representing a clinically relevant mechanism linking pharmacological burden to digestive symptoms.

Digestive disturbances were reported in 50.9% of participants and were significantly associated with the number of medications per patient, with each additional drug corresponding to approximately 23% higher odds of gastrointestinal symptoms. While this association highlights the potential impact of pharmacological burden on gastrointestinal homeostasis, it should be interpreted cautiously. Reverse causation is plausible, as patients experiencing gastrointestinal symptoms may accumulate medications prescribed for their management, such as laxatives or acid-suppressive agents, thereby increasing the total medication count.

Nevertheless, drug-induced effects particularly from anticholinergics, antidepressants, and antiparkinsonian agents may contribute to gastrointestinal dysfunction by slowing intestinal transit, reducing salivary secretion, or causing mucosal irritation (51–53). Although the present study did not quantify anticholinergic burden using standardized scales, the high prevalence of medications with known anticholinergic effects suggests that this mechanism may have contributed to the observed gastrointestinal disturbances. These manifestations not only compromise comfort and quality of life but also contribute to reduced food intake, nutrient malabsorption, and anorexia, further aggravating malnutrition in individuals already at high nutritional risk (54, 55). Accordingly, the observed relationship likely reflects a complex and bidirectional interplay between medication exposure and gastrointestinal symptomatology, rather than a unidirectional causal pathway.

Beyond medication-related mechanisms, gastrointestinal dysfunction in dementia may arise from multifactorial processes, including autonomic dysregulation, alterations in the gut–brain axis, and reduced physical activity, all of which can impair gut motility and digestion (56, 57). Emerging evidence also suggests that gut dysbiosis and neuroinflammatory pathways may contribute to both gastrointestinal and cognitive symptoms, reinforcing a cycle of inflammation, malnutrition, and frailty (58, 59).

Unexpectedly, no significant association was observed between polypharmacy and anthropometric parameters (BMI, MNA^®^, MAMA, CC), although a weak positive correlation with HGS was identified. This finding should be interpreted cautiously and is more plausibly explained by survivor bias or deprescribing practices in advanced disease stages, whereby more functionally impaired patients may receive fewer medications, rather than reflecting a beneficial effect of higher medication burden on muscle strength.

Given the very small proportion of participants classified as non-malnourished according to MNA^®^ and GLIM criteria, a meaningful stratified analysis of medication profiles across nutritional categories was not feasible. In addition, the extremely limited number of participants not exposed to polypharmacy precluded robust comparisons between polypharmacy and non-polypharmacy groups. Polypharmacy thus appears to be a pervasive characteristic of this population at admission, largely independent of nutritional classification.

A further noteworthy observation was the mismatch between clinical diagnosis and medication use: despite the absence (or very low frequency) of formally documented Parkinsonian diagnoses, 16 participants were receiving antiparkinsonian therapy. This likely reflects overlapping or under-characterized syndromes, such as dementia with Lewy bodies, vascular parkinsonism, or Parkinson’s disease dementia (51–53). These diagnostic challenges underscore the importance of comprehensive and interdisciplinary assessment at the time of institutional admission.

### 4.4 Clinical implications

The convergence of advanced age, cognitive decline, malnutrition, sarcopenia, polypharmacy, and digestive disturbances generates a compounded risk profile requiring coordinated multidisciplinary care. Identifying several simple markers, such as MAMA or CC (for lean mass) and HGS (for functionality), may enable timely interventions to prevent complications, such as falls, pressure injuries, and infections. Evidence supports the effectiveness of combined strategies, including individualized high-protein supplementation, resistance exercise tailored to cognitive level, and medication review to minimize drug–nutrient interactions (50, 59–62). Nutritional interventions in dementia should not focus solely on caloric intake but integrate muscle preservation, inflammation control, and medication optimization, aiming to preserve autonomy and quality of life.

### 4.5 Strengths and limitations

The strengths of this multicenter study include its comprehensive and clinically relevant multidimensional approach, integrating anthropometry, functional assessment, and validated nutritional tools in a real-world setting. The inclusion of polypharmacy as a key variable further enhances the understanding of pharmacological burden in institutionalized individuals with dementia.

This study provides evidence of a very high prevalence of malnutrition, sarcopenia, and digestive disturbances among patients with dementia at the time of admission to long-term care units. The coexistence of advanced cognitive decline, functional dependency, and extensive polypharmacy underscores a state of compounded vulnerability that may adversely affect nutritional and functional outcomes. Anthropometric and functional assessments, particularly CC, MAMA, and HGS, proved central for capturing both structural and performance-related deficits. In addition, digestive symptoms, reported by most participants, emerged as a clinically relevant manifestation of the combined pharmacological and disease-related burden.

These findings support the need for systematic, multidomain screening strategies integrating nutritional, functional, and gastrointestinal assessment alongside comprehensive medication review. Early, individualized, and interdisciplinary interventions targeting nutrition, sarcopenia, and digestive health may help preserve autonomy, reduce complications, and improve quality of life in institutionalized individuals with dementia.

Despite these strengths, several limitations must be acknowledged. First, the cross-sectional design precludes causal inference regarding the relationships among cognitive decline, malnutrition, sarcopenia, and digestive symptoms. Second, muscle mass was estimated using indirect anthropometric measures (CC and MAMA) rather than gold-standard techniques such as DXA, BIA, or CT scan. While anthropometric methods are validated and feasible in geriatric and institutional settings, they may be influenced by factors such as edema or age-related changes in tissue composition, potentially introducing measurement bias (8, 18).

Third, the operationalization of the GLIM criteria represents an important methodological consideration. Dementia was considered to fulfill the etiologic criterion of disease burden/inflammation for all participants, meaning that malnutrition classification relied primarily on phenotypic criteria. This approach may have increased sensitivity at the expense of specificity, potentially contributing to higher prevalence estimates. In addition, data on reduced food intake were not systematically available, precluding sensitivity analyses using alternative etiologic criteria. This limitation reflects the real-world clinical context of heterogeneous referral sources and inconsistent caregiver reporting, and limits comparability with studies using stricter GLIM definitions. Nevertheless, MNA^®^ results present malnutrition/risk of malnutrition similar to those obtained with GLIM.

Fourth, due to the particular clinical setting, the handgrip strength assessment protocol deviated from EWGSOP2 recommendations, as only the first valid attempt was systematically recorded. The reported values should therefore be interpreted as reflecting standardized initial effort rather than maximal strength.

Fifth, the evaluation of micronutrients (e.g., vitamin D, vitamin B12) and biochemical markers (e.g., albumin, thyroid function) was not feasible, limiting a more comprehensive characterization of nutritional and metabolic status. Sixth, dementia subtype classification was frequently unavailable, precluding etiological comparisons (63).

Seventh, medication burden was assessed primarily using total medication count, which does not capture qualitative pharmacological effects. In particular, anticholinergic burden was not quantified using a validated scale, limiting the ability to assess its independent contribution to gastrointestinal symptoms. In addition, medication indication was not consistently available, precluding sensitivity analyses excluding drugs prescribed for gastrointestinal symptom management. Medication data were derived from prescribing and administration records rather than direct verification of intake, which may not fully account for non-adherence or recent medication changes prior to admission.

Finally, although clinically relevant, cognitive (MMSE) and nutritional (MNA^®^) variables were not included simultaneously in regression models to avoid multicollinearity and model overfitting. Moreover, the absence of stratified analyses by dementia severity (e.g., GDS stage) and the lack of direct assessment of deprescribing practices limit further exploration of potential survivor bias.

### 4.6 Future directions

Future research should adopt longitudinal designs to clarify the temporal dynamics between polypharmacy, nutritional decline, and dementia progression. Incorporating validated scales to quantify anticholinergic and sedative burden, alongside detailed pharmacological profiling, will be essential to identify specific drug classes that may drive gastrointestinal dysfunction and malnutrition. Furthermore, interventional trials combining personalized nutritional support, targeted resistance exercise, and medication optimization strategies are warranted to determine the most effective multidomain approaches for preserving function and improving quality of life in this highly vulnerable population.

## 5 Conclusion

This multicenter study demonstrates a very high burden of malnutrition, sarcopenia, polypharmacy, and digestive disturbances among patients with dementia at admission to long-term care units. Anthropometric and functional measures, particularly MAMA, CC, and HGS, provide complementary insights into structural and functional decline. A higher prescribed medication burden was independently associated with digestive disturbances, supporting the clinical relevance of medication review as part of multidomain nutritional and functional assessment.

These findings highlight the importance of implementing systematic, multidomain screening strategies at institutional admission, enabling early, individualized, and interdisciplinary care aimed at improving clinical outcomes and quality of life in this vulnerable population.

## Data Availability

The original contributions presented in this study are included in the article/Supplementary material, further inquiries can be directed to the corresponding author.
