# Supplementary material for: Nutrition, functional status, polypharmacy, and digestive disturbances in institutionalized patients with dementia: insights from comprehensive screening at admission
Source: Front Nutr. 2026 Jul 2;13:1779867. doi: 10.3389/fnut.2026.1779867 (PMC13372590; doi:10.3389/fnut.2026.1779867)
Supplement: Supplementary file 1 [file Table_1.docx]

**Supplementary Table S1.** Multivariable linear regression models for anthropometric indicators of muscle mass (MAMA and CC)

| Outcome | Predictor | B | SE | β | t | p-value | 95% CI (B) | VIF |
| --- | --- | --- | --- | --- | --- | --- | --- | --- |
| MAMA | (Constant) | -18.77 | 6.13 |  | -3.06 | 0.003 | [-30.93, -6.61] |  |
| ($\boldsymbol{R}^{\boldsymbol{2}}\boldsymbol{=0.44}$) | Male sex | 6.31 | 1.15 | 0.43 | 5.48 | **<0.001** | [4.03, 8.59] | 1.05 |
|  | BMI | 1.16 | 0.17 | 0.52 | 6.64 | **<0.001** | [0.81, 1.50] | 1.05 |
|  | *Excluded:* | *Age, HGS, SARC-F* |  |  |  |  |  |  |
| CC | (Constant) | 8.87 | 3.23 |  | 2.75 | 0.007 | [2.46, 15.28] |  |
| ($\boldsymbol{R}^{\boldsymbol{2}}\boldsymbol{=0.51}$) | BMI | 0.70 | 0.05 | 0.71 | 12.75 | **<0.001** | [0.59, 0.81] | 1.02 |
|  | Male sex | 1.06 | 0.40 | 0.15 | 2.68 | **0.009** | [0.27, 1.84] | 1.02 |
|  | *Excluded:* | *Age, HGS, SARC-F* |  |  |  |  |  |  |

*Note: B, unstandardized coefficient; SE, standard error; β, standardized coefficient; CI, confidence interval; VIF, variance inflation factor; MAMA, mid-arm muscle area; CC, calf circumference; BMI, body mass index.*

**Supplementary Table S2.** Spearman Correlation Matrix between anthropometric and functional variables

|  | BMI | MUAC | TSF | MAMA | CC | HGS | SARC-F |
| --- | --- | --- | --- | --- | --- | --- | --- |
| BMI | 1.00 | 0.79* | 0.46* | 0.69* | 0.70* | 0.10 | -0.11 |
| MUAC |  | 1.00 | 0.57* | 0.89* | 0.68* | 0.09 | -0.14 |
| TSF |  |  | 1.00 | 0.15 | 0.35* | -0.19 | 0.12 |
| MAMA |  |  |  | 1.00 | 0.61* | 0.21** | -0.23** |
| CC |  |  |  |  | 1.00 | 0.24** | -0.24** |
| HGS |  |  |  |  |  | 1.00 | -0.13 |
| SARC-F |  |  |  |  |  |  | 1.00 |

*Note: BMI, body mass index; MUAC, mid-upper arm circumference; TSF, triceps skinfold; MAMA, mid-arm muscle area; CC, calf circumference; HGS, handgrip strength. * Correlation is significant at the 0.01 level (2-tailed). ** Correlation is significant at the 0.05 level (2-tailed).*

**Supplementary Table S3.** Multivariable logistic regression model for the presence of digestive symptoms

| Predictor | B | SE | Wald | p-value | OR | 95% CI (OR) |
| --- | --- | --- | --- | --- | --- | --- |
| Number of medications | 0.207 | 0.063 | 10.99 | **0.001** | 1.23 | [1.09, 1.39] |
| Constant | -1.583 | 0.575 | 7.57 | 0.006 | 0.20 |  |
| *Excluded variables:* | *Nutritional status (GLIM criteria), Dementia stage (GDS)* |  |  |  |  |  |

*Model Fit Indices: Omnibus Test p < 0.001; Nagelkerke* $R^{2}=0.182$*; Hosmer-Lemeshow Test p = 0.117. OR, Odds Ratio; CI, confidence interval.*
